# Insights into the reproduction of some Antarctic dendroceratid, poecilosclerid, and haplosclerid demosponges

**DOI:** 10.1371/journal.pone.0192267

**Published:** 2018-02-08

**Authors:** Vasiliki Koutsouveli, Sergi Taboada, Juan Moles, Javier Cristobo, Pilar Ríos, Andrea Bertran, Joan Solà, Conxita Avila, Ana Riesgo

**Affiliations:** 1 Department of Life Sciences (Invertebrate Division), The Natural History Museum of London, London, United Kingdom; 2 Department of Evolutionary Biology, Ecology, and Environmental Sciences, Faculty of Biology, and IRBio (Biodiversity Research Institute), University of Barcelona, Barcelona, Catalonia, Spain; 3 Instituto Español de Oceanografía (IEO), Gijón, Spain; Laboratoire de Biologie du Développement de Villefranche-sur-Mer, FRANCE

## Abstract

Sponges are a dominant element of the Antarctic benthic communities, posing both high species richness and large population densities. Despite their importance in Antarctic ecosystems, very little is known about their reproductive patterns and strategies. In our study, we surveyed the tissue of six different species for reproductive elements, namely, *Dendrilla antarctica* Topsent, 1905 (order Dendroceratida), *Phorbas areolatus* (Thiele, 1905), *Kirkpatrickia variolosa* (Kirkpatrick, 1907), and *Isodictya kerguelenensis* (Ridley & Dendy, 1886) (order Poecilosclerida), and *Hemigellius pilosus* (Kirkpatrick, 1907) and *Haliclona penicillata* (Topsent, 1908) (Haplosclerida). Samples of these six species containing various reproductive elements were collected in Deception Island and were processed for both light and transmission electron microscopy (TEM). Even though we were not able to monitor the entire reproductive cycle, due to time and meteorological conditions, we report important aspects of the reproduction of these species. This includes oocyte and embryo morphology and cell ultrastructure, follicular structures and nurse cell activity, as well as vitellogenesis. All species were brooding their embryos within their mesohyl. Both oocytes and embryos were registered in the majority of the studied species, and a single sperm cell being carried to an egg for fertilization was observed in *H*. *penicillata*. While the reproductive periods of all species coincided temporally, some of them seemed to rely on a single spawning event, this being suggested by the synchronic oogenesis and embryogenesis occurrence of *D*. *antarctica*, *P*. *areolatus* and *I*. *kerguelenensis*. In contrast, *K*. *variolosa* had an asynchronous embryo development, which suggests several larval release events. Our results suggest that differences in the reproductive strategies and morphological traits might succeed in the coexistence of these species at the same habitat avoiding the direct competition between them.

## Introduction

Sponges are a dominant element of the Antarctic benthic communities, posing both high species richness (more than 300 species) and large population densities [1,2,3,4]. Among the Antarctic representatives of the phylum Porifera, 81% are Demospongiae, of which almost half belong to the order Poecilosclerida and 17% to the order Haplosclerida [2], finding a high percentage of endemism among them [4]. Antarctic sponges comprise three-dimensional communities that provide habitat, refuge and favour recruitment to many other invertebrate species [4]. The recruitment and growth patterns of several Antarctic sponges have been recently reported in McMurdo Sound (Ross Sea) specimens. A period of 30 year-stasis, with virtually no recruitment and very slow growth, followed by massive settlement events was reported in the last 10 years [5]. Interestingly, the multi-species massive recruitment was observed during periods of heavy ice cover, which could potentially protect the coast from wave disturbance, thus enhancing the transport of sponge propagules [5]. However, larvae production, fitness, and their potential dispersal capabilities, which may provide the grounds for sponge recruitment, are poorly studied in warm and temperate oceans, and even less in the Southern Ocean. Indeed, the reproductive and/or dispersal capabilities of the sponges inhabiting Antarctic rocky bottoms have only been addressed briefly for a handful of species [6,7] and in more detail in only two species, *Stylocordyla chupachups* (as *S*. *borealis* [8]) and *Mycale (Oxymycale) acerata* [9]. The environmental parameters which drive the reproduction of Antarctic sponges are unknown. However, the reproductive behaviour is considered to be triggered by environmental stimuli other than temperature in the constantly cold waters of the North Atlantic deep sea, such as primary productivity blooms [10,11,12]. This has never been investigated for Antarctic sponges.

Among the phylum Porifera, the class Demospongiae presents the greatest variety in reproductive processes. They possess four larval types including clavablastula, dispherula, hoplitomella, and parenchymella [13,14], being the last one the most abundant larval type, found in the orders Dendroceratida, Haplosclerida and Poecilosclerida, among others). Parenchymella larvae could be the result of both internal and external fertilization in oviparous and ovoviviparous demosponges and its development proceeds with total chaotic cleavage for the formation of a morula (see [15] for a review). In this study, we examined the tissue of six species of the orders Dendroceratida (*Dendrilla antarctica*), Poecilosclerida (*Phorbas areolatus*, *Kirkpatrickia variolosa*, and *Isodyctia kerguelenensis*) and Haplosclerida (*Hemigellius pilosus* and *Haliclona penicillata*) for reproductive elements. Even though all three taxa possess parenchymella larvae, their development differ from one to another: members of the order Dendroceratida have isolecithal eggs without any polarization; those in Poecilosclerida have in turn a telolecithal and polarized egg that undergoes total cleavage, either equal or unequal, while members of the order Haplosclerida are characterized by eggs with a great amount of phagocyted trophocytes which form yolk granules during cleavage [15,16]. Although oviparity is the most widespread reproductive mode in members of all the above-mentioned orders with parenchymella larvae, oviviparous or viviparous species also appear within these groups [17]. Such different reproductive types have been observed to vary among orders as consequence of adaptation processes to environmental conditions without any phylogenetic signal [17].

Several features, such as the size of the egg and embryo, the vitellogenesis and the cleavage patterns, are linked to the different reproductive strategies of an animal group [9,18]. The increase in size of the egg is thought to be the result of transition from oviparity to ovoviviparity, which is coupled with the change from small oligolecithal to bigger polylecithal eggs respectively [18]. The cleavage can be equal or unequal, producing either equally-sized blastomeres or macro- and micromeres that will undergo different fates in the larva [15]. Another differential feature during development is the yolk nature during oocyte maturation and embryogenesis, which might be protein, lipid or glycogen. It can be derived directly from the embryo (e.g. through Golgi apparatus and direct phagocytosis of other particles or cells) or can be produced by nurse cells, situated either inside or outside the embryo close to the follicular membrane, or both [19,20]. This has ecological implications for the duration of both gametogenesis and embryogenesis. While some specific adaptations in the reproductive patterns of marine invertebrates living in extreme cold conditions have been reported [8], Riesgo and collaborators [9] found that the embryos of the Antarctic *Mycale (Oxymycale) acerata* had much higher content of lipid yolk than its tropical counterpart *Mycale (Mycale) laevis*, suggesting that it may represent an adaptation to extreme cold conditions. Whether this is a general trend in Antarctic sponges is still unknown.

Our aim in this study was to describe and compare the ultrastructure of female reproductive features of six demosponge species from different taxonomic groups co-occurring in the same habitat in Antarctica, to test whether they show similar adaptations in their vitellogenesis or morphological features to those observed in *M*. *acerata* [9]. Oocyte and embryo morphology and their cell ultrastructure, follicular structures and nurse cell activity as well as vitellogenesis processes were evaluated.

## Material and methods

### Sample collection and preservation

**Fig 1:**
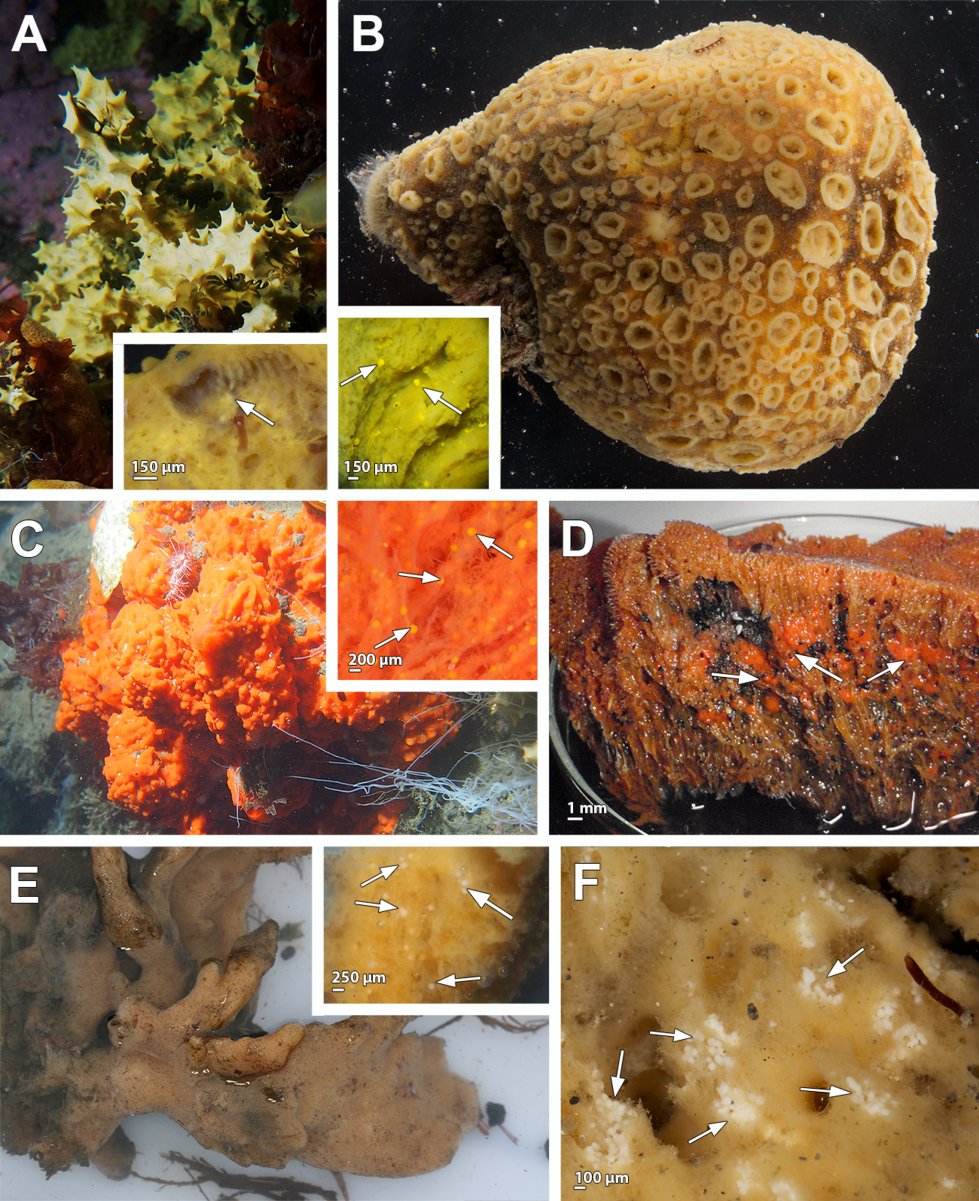
Pictures of live specimens and their embryos within the tissue. **A**. *Dendrilla antarctica*. Insert: yellow embryos (white arrows). **B**. *Phorbas aerolatus*. Insert: bright yellow embryos (white arrows). **C**. *Kirkpatrickia variolosa*. Insert: large amount of bright orange embryos (white arrows). **D**. *Isodictya kerguelenensis* specimens in which big red embryos dispersed through the mesohyl (white arrows). **E.** *Hemigellius pilosus*. Insert: embryos scattered throughout the mesohyl (white arrows). **F.** *Haliclona penicillata* showing embryos contained in brooding chambers, each of them with approximately 30–60 embryos (white arrows).

Samples of *Dendrilla antarctica*, *Phorbas areolatus*, *Kirkpatrickia variolosa*, *Isodictya kerguelenensis*, *Hemigellius pilosus* and *Haliclona penicillata* (Fig 1; Table 1) were collected by SCUBA diving on rocky outcrops at 15 m depth in Fildes Point and Whalers Bay, Deception Island (62°59′19,3″S, 60°33′29,1″W and 62°59′23,7″S, 60°33′40,9″W, respectively; South Shetland Islands, Antarctica) during January 14^th^-27^th^ 2011, February 2^nd^-20^th^ 2013, and January 6^th^-February 4^th^ 2016. A small portion of tissue from three to five specimens per species was collected, although not all of them showed reproductive features (Table 1). Permits for collection of marine invertebrates were issued by the Spanish Ministry of Science and Innovation (CPE-EIA-2011-7 (for 2011 and 2013 samples), and CPE-EIA-2015-7 (for 2016 samples)). For each specimen collected, a portion was preserved for light microscopy (in 4% formalin buffered in seawater) and for transmission electron microscopy (TEM) (in 2.5% glutaraldehyde in PBS), according to protocols described in [21].

**Table 1:** Details on the number of reproductive specimens per species and the type of reproductive elements found in the tissue.

Order, Family, Species	Sampling	# individuals	# individuals with reproductive elements	Type of reproductive elements
Dendroceratida				
Darwinellidae *Dendrilla antarctica* Topsent, 1905	February 2013 and January 2016	5	4	oocytes/embryos
Poecilosclerida				
Hymedesmiidae *Phorbas areolatus* (Thiele, 1905)	February 2013 and 2016	4	4	embryos
*Kirkpatrickia variolosa* (Kirkpatrick, 1907)	February 2013 and January 2016	3	2	oocytes/embryos
Isodictyiidae *Isodictya kerguelenensis* (Ridley & Dendy, 1886)	January 2011	3	1	embryos
Haplosclerida				
Niphatidae *Hemigellius pilosus* (Kirkpatrick, 1907)	February 2013	3	3	oocytes/embryos
Chalinidae *Haliclona penicillata* (Topsent, 1908)	February 2013	3	2	oocytes/embryos

### Sample processing and measurements

Samples preserved for light microscopy were rinsed for 2h in distilled water and dehydrated through an ascending series of ethanol (70%, 96%, 100%) and xylene. Samples were then embedded in paraffin at 60°C overnight and cut with a Microtome Micron HM325 to 5 μm sections. Staining was performed using both Methylene blue and Hematoxilin-Eosin standard protocols. Pictures of reproductive elements were obtained with a microscope Olympus BX43 and an SC50 5MP colour CMOS camera. The counting and measurements (maximum diameter) of reproductive elements in each section were performed with the CellSens image analysis software of Olympus.

Samples preserved for TEM were subjected to a protocol of rinsing, fixation, dehydration and embedding following [9]. The sections of Spurr resin blocks were performed at 64 nm using an ULTRACUT ultramicrotome, stained with lead citrate and uranyl acetate. They were observed with a JEOL 1010 electron microscope with a Gatan module for image digitalization at the Microscopy Unit at the Scientific and Technological Centres, Universitat de Barcelona (CCiT-UB).

## Results

We examined the reproductive elements of six different Antarctic demosponges, for which the oocytic and embryonic ultrastructural features are described in detail for the first time here.

### Oocyte and embryonic features of *Dendrilla antarctica* (Demospongiae, Dendroceratida, Darwinellidae)

**Fig 2:**
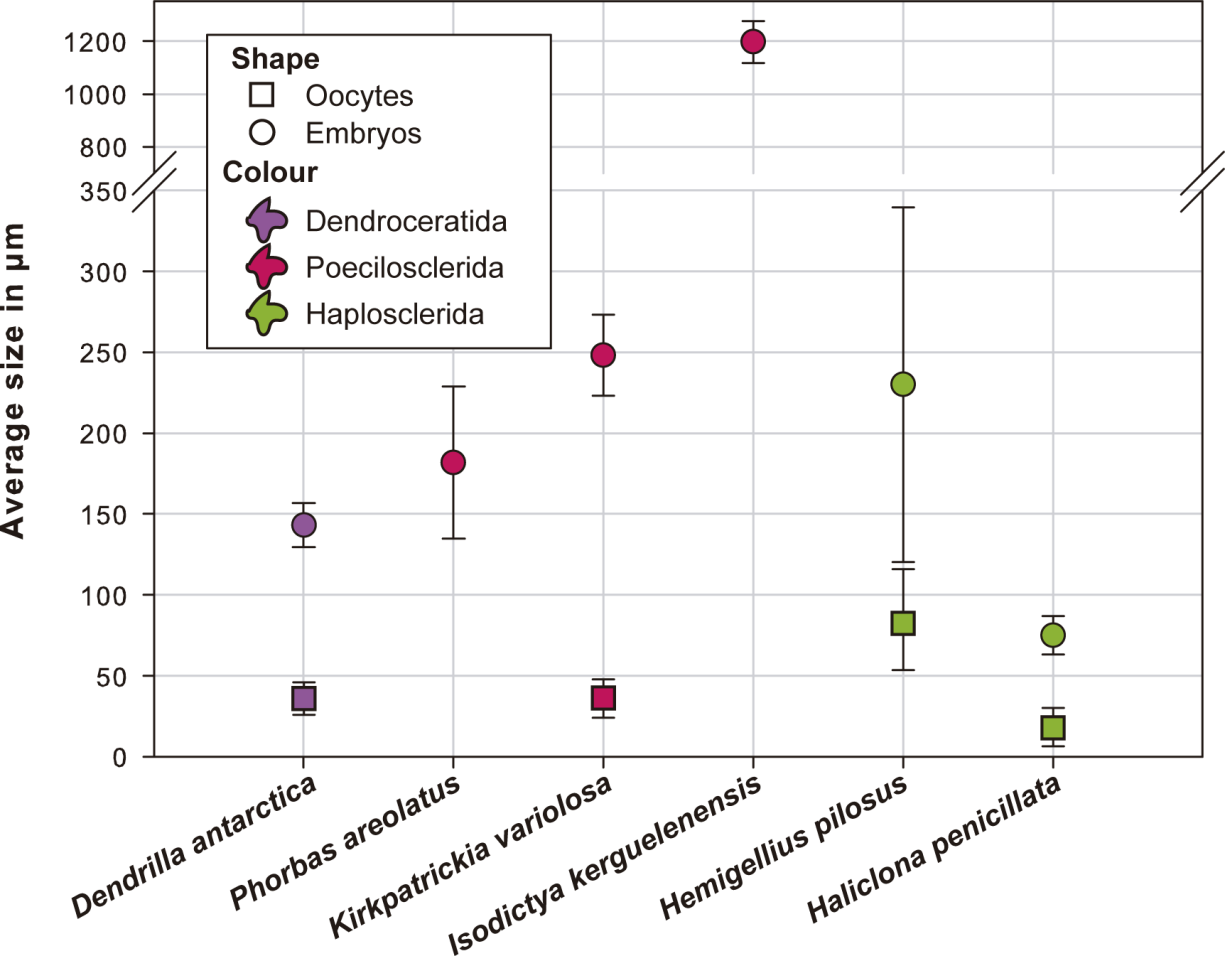
Average size (μm) and standard deviation of oocytes (squares) and embryos (circles) found in the different studied species. Each colour represents a different order: violet is Dendroceratida, pink is Poecilosclerida and green is Haplosclerida.

**Fig 3:**
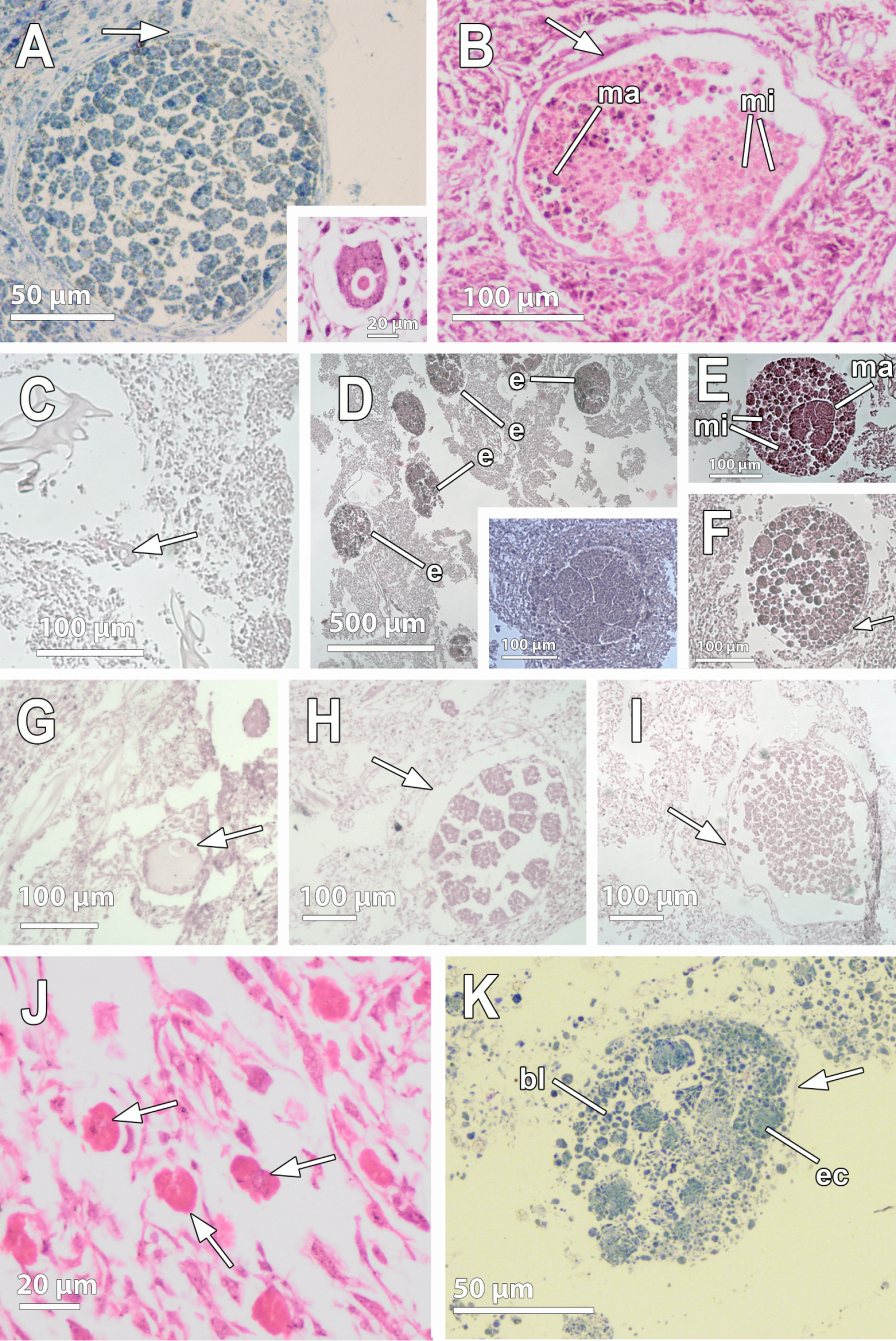
Light microscopy observations of reproductive elements in Antarctic species. **A.** Embryo of *Dendrilla antarctica* situated below the pinacoderm and surrounded by a follicle membrane (white arrow). All cells within the embryo have polygonal morphology, with those in the periphery more flattened than internal ones. The oocyte (insert) is also surrounded by a thin cellular follicle. **B.** Embryo of *Phorbas aerolatus* showing both micromeres (mi) and macromeres (ma) in the posterior and the anterior part, respectively. The follicle layer is also shown (white arrow). **C.** Small previtellogenic oocyte of *Kirkpatrickia variolosa* observed close to a canal (white arrow). **D.** Several mid- and late-stage embryos (e) of *K*. *variolosa* close to or in the exhalant canals, and early-stage embryos scattered in the mesohyl (insert). **E-F.** Mid- stage (E) and late-stage (F) embryos of *K*. *variolosa*, showing micromeres (mi) and anterior macromeres (ma). Embryo surrounded by a follicle layer (white arrow). **G.** Pre-vitellogenic oocyte of *Hemigellius pilosus* close to a canal, showing engulfment of nurse cells (white arrow). **H.** Early embryo of *H*. *pilosus* surrounded by a follicle (white arrow). Note the space between the follicle and the embryo. **I**. Late embryo of *H*. *pilosus*, surrounded by a follicle (white arrow). **J.** Vitellogenic oocytes of *Haliclona penicillata* without a clear follicle. **K.** Embryo of *H*. *penicillata* showing blastomeres (bl) and engulfed amoeboid cells inside (ec). Follicle is observed (white arrow).

**Fig 4:**
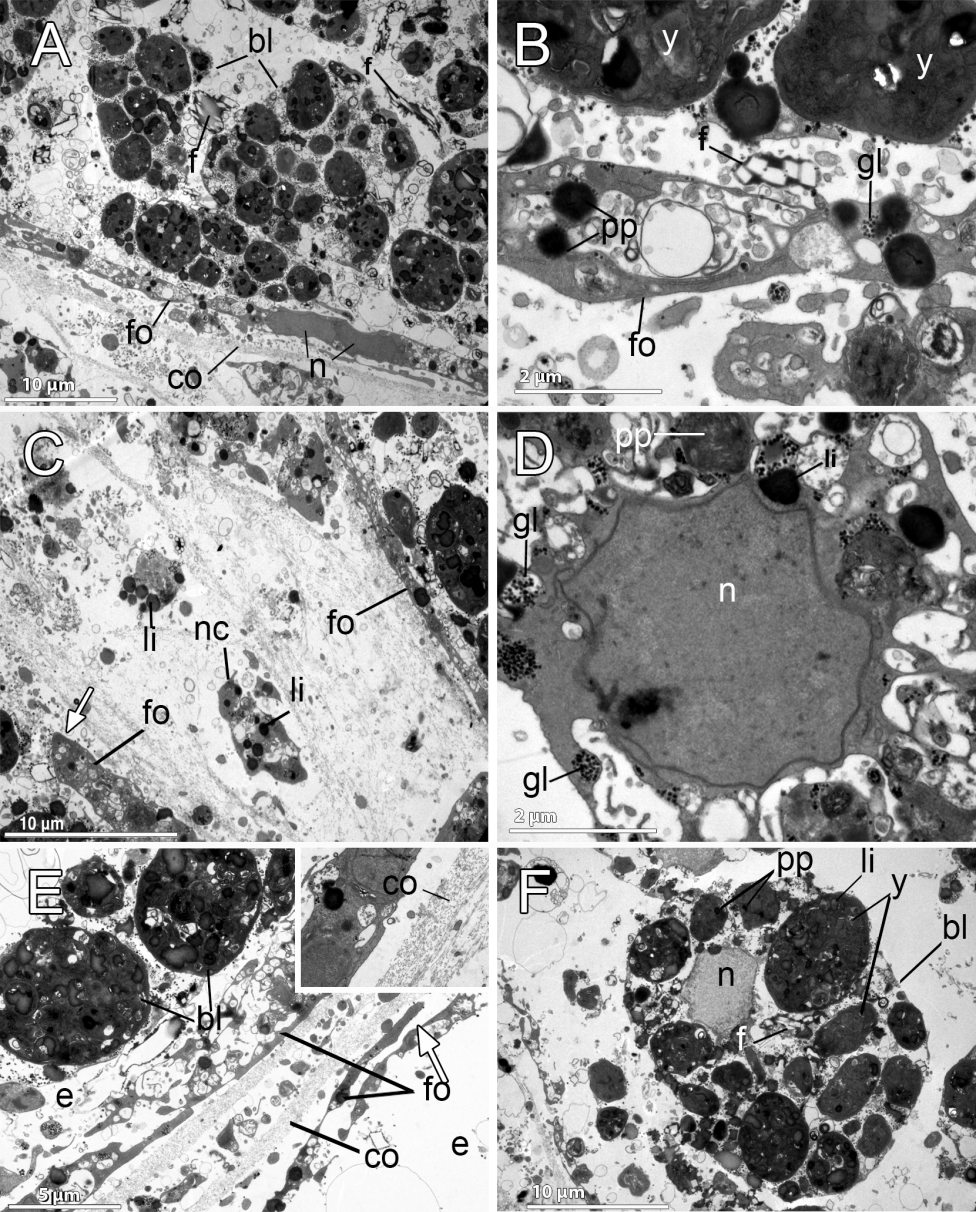
Ultrastructural features of the reproductive elements of *Dendrilla antarctica*. **A.** Embryonic blastomeres (bl) with spongin fibers (f) interspersed. The follicle (fo) contains elongated cells, probably syncitial (sy). **B.** Detail of the follicle cell (fo) showing protein yolk platelets (pp), glycogen (gl), and embryonic blastomeres with heterogenous yolk (y). Exchange activity between them is observed in the several endocytotic processes. Spongin fibers (f) in the periphery of the embryo. **C.** Nurse cells (nc) within the mesohyl close to the periphery of the embryo, containing lipid (li) yolk. Note the follicle cells (fo) surrounding the embryo. **D.** Nurse cell, showing the nucleus (n), lipid yolk (li), heterogeneous protein platelets (pp), and glycogen (gl) within the cytoplasm. **E.** Two embryos (e) with their follicle layers (fo). Below each follicle, there is a thick layer of collagen (co). Blastomeres (bl) of one embryo are observed. Openings of the follicle (fo) indicated with a white arrow. **Inset.** Close up of the collagen (co) layer surrounding the follicle. **F.** Blastomere (bl) showing the nucleus (n) and spongin fibers (f), electron-dense heterogeneous yolk platelets (y) of both protein (pp) and lipid (li) nature.

Four out of the five specimens collected from *D*. *antarctica* species were reproductive, two of them showing oocytes and the other two with embryos (Table 1, Fig 1A, insert). The oocytes in both specimens were in the same reproductive stage, being isolecithal, vitellogenic, with 34 μm average max. diameter (Fig 2) and surrounded by a thin cellular follicle (Fig 3A, insert), leaving a space within the follicle (Fig 3A, insert). Oocytes were located close to the canals while bright yellow embryos were distributed across the whole body of *D*. *antarctica*, although they appeared most abundantly within the first 5 cm below the pinacoderm (Figs 1A and 3A). In the embryos, cleavage was total and equal (not shown). Cleaving embryos were found (approx. 140 μm in maximum diameter, Fig 2) in similar stages and surrounded by a follicle (Fig 3A). The follicle enveloping each cleaving embryo, consisted of a relatively thick layer of collagen (2 μm) and a single layer of flattened cells (Fig 4A–4C and 4E). Follicle cells were elongated and narrow (ca. 4 μm in width), and showed two nuclei (Fig 4A). In some parts of the follicle, the tips of the follicle cells were not in contact, leaving a space between them (Fig 4C and 4E). An intense traffic of nutrients/particles across the follicle, with several endocytic processes, was observed (Fig 4B and 4C). The nurse cells were amoeboid and full of lipid and protein yolk and were located within the mesohyl close to the follicle cells (Fig 4C and 4D). They were medium-sized cells of ca. 5 μm in largest diameter (Fig 4C).

**Fig 5:**
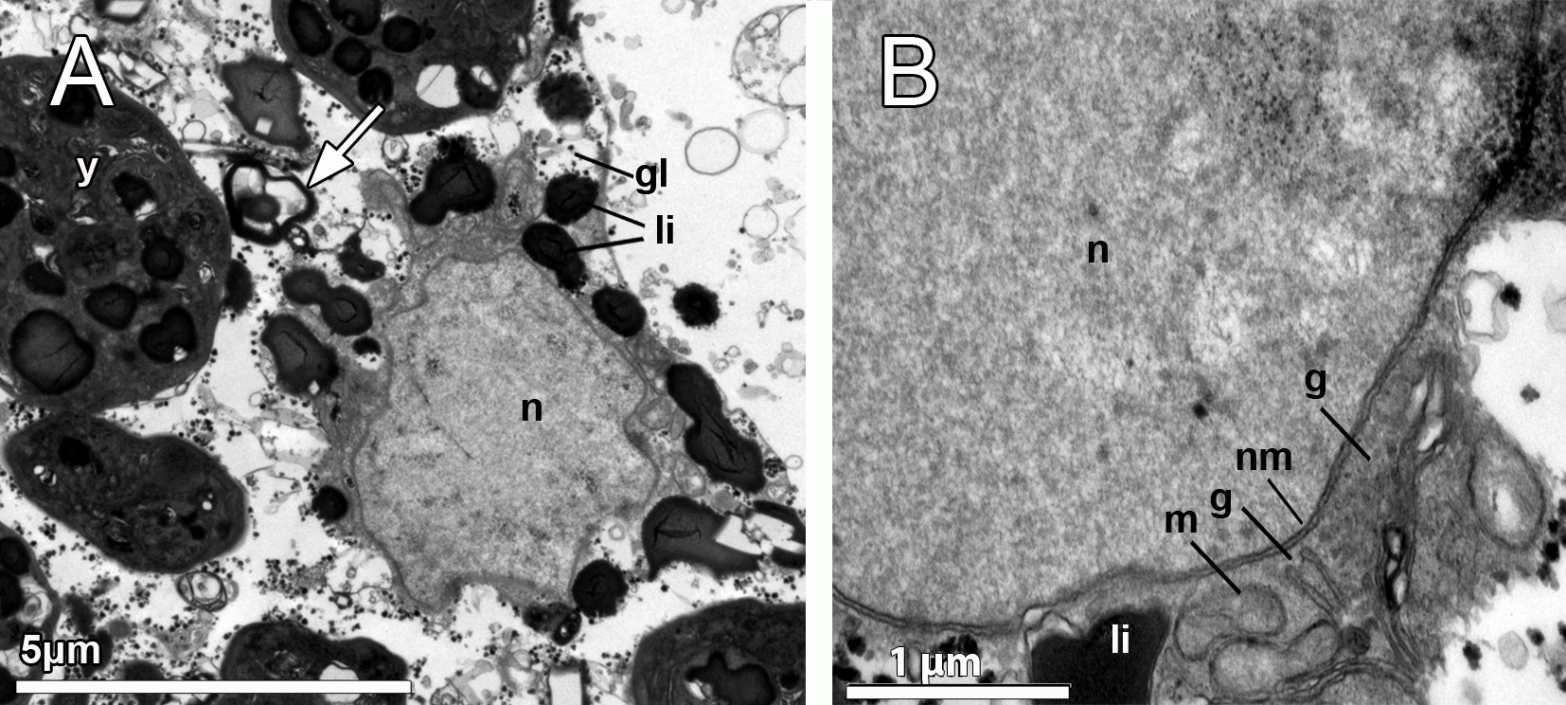
Yolk formation in the embryo of *Dendrilla antarctica*. **A-B.** Blastomere showing the double membrane (nm) of the nucleus (n), glycogen (gl), heterogeneous yolk platelets (y), spongin fibers (white arrow), and highly electron-dense lipid droplets (li). Note the smaller lipid yolk platelets (li) close to the nucleus, where the well-developed Golgi apparatus (g) and mitochondria (m) are also observed.

The blastomeres in the embryos were up to 20–30 μm in diameter, being the peripheral slightly more flattened than the internal ones, which showed a polygonal morphology (Figs 3A and 4A). They possessed a non-nucleolated nucleus of ca. 5 μm and several large compound yolk platelets (5–7 μm) within the cytoplasm (Fig 4A, 4E and 4F). The yolk platelets were highly heterogeneous, with some electron-dense material, suggesting a protein nature, but also with content similar to lipid yolk (Figs 4A, 4E, 4F and 5A). The blastomeres contained several mitochondria and a well-developed Golgi apparatus (Fig 5B), glycogen and lipid and protein yolk platelets (Fig 4F). The Golgi was found actively forming lipid yolk platelets that were initially small and located close to the nucleus (Fig 5A and 5B). Spongin fibres at the periphery of the embryo (Fig 4B) and also within the embryo among the blastomeres were observed (Fig 4F). No bacteria could be detected in the embryos, but few scattered bacterial cells were observed within the mesohyl (not shown).

#### *Embryonic features of* Phorbas areolatus *(Demospongiae*, *Poecilosclerida*, *Hymedesmiidae)*

**Fig 6:**
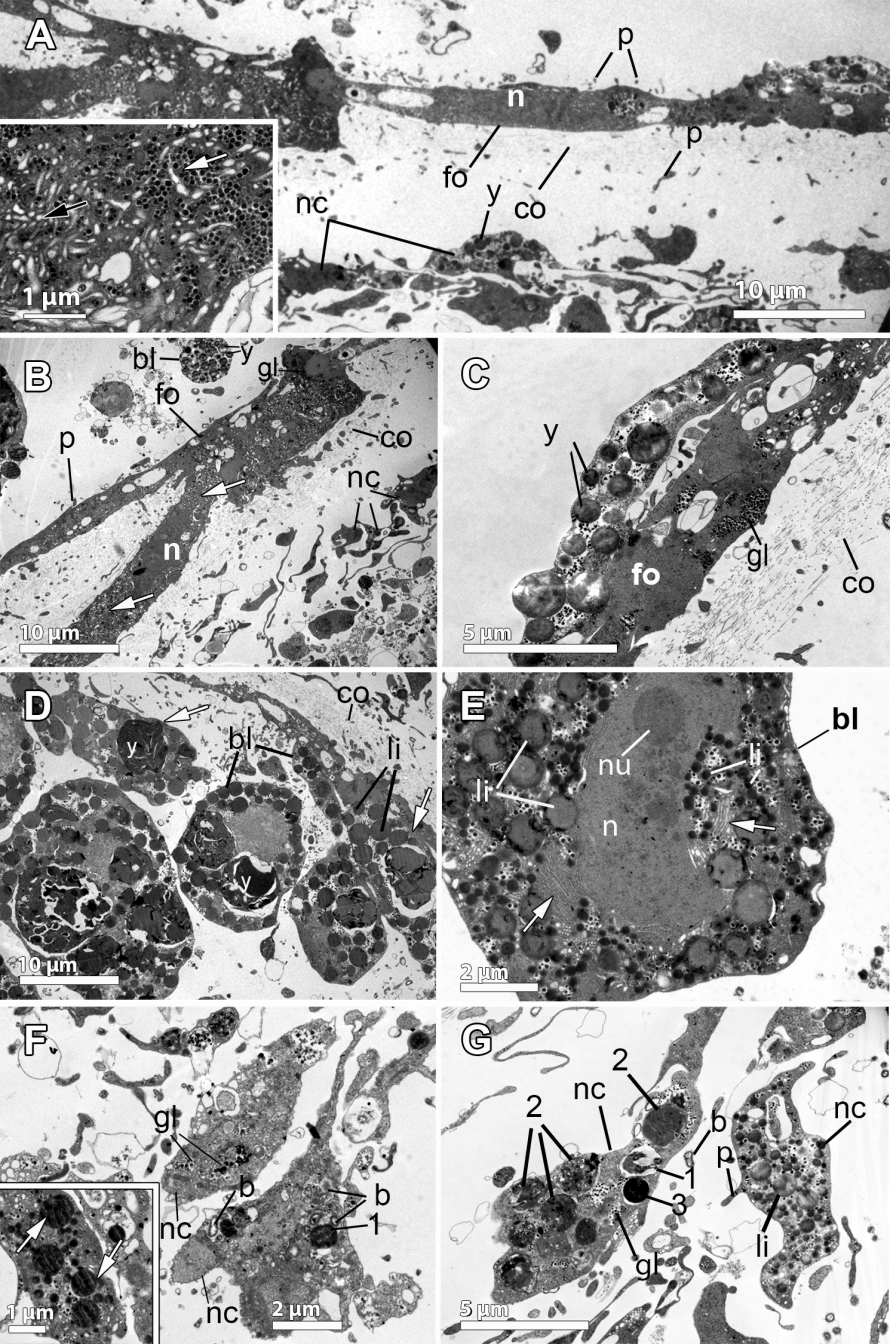
Ultrastructural features of the embryo of *Phorbas aerolatus*. **A-B.** Follicle cell (fo) surrounding the embryo (bl) containing large endoplasmic reticulum (white arrows), and the collagen layer (co) developed below the follicle. Note the nucleus (n) and the multiple cytoplasmic processes (p) of the follicle cell formed towards the embryo and the mesohyl. Amoeboid nurse cells (nc) in the mesohyl with heterogeneous yolk (y) similar to the yolk within blastomeres (y). **Inset.** Well-developed smooth endoplasmic reticulum (black arrow) and glycogen (white arrow). **C.** Detail of a follicle cell (fo) containing glycogen (gl) and yolk granules (y). Thick layer of collagen (co) in the outer part. **D.** Detail of the periphery of the embryo showing blastomeres (bl) of different sizes, being the ones in the outer part smaller and more flattened (white arrows). Blastomeres full of lipid yolk (li) and large heterogeneous yolk platelets (y). **E.** Detail of blastomere containing a large nucleolated (nu) nucleus (n), glycogen (gl), and lipid droplets (li). Note the well-developed Golgi apparatus (white arrows). **F-G.** Nurse cells (nc) in detail showing multiple pseudopodia (p), lipid (li) droplets, and glycogen (gl). Note the different stages of formation of the heterogeneous protein yolk platelet (1 less mature to 3 completely formed), which are the result of the digestion of bacteria (b). **Inset in F.** Nurse cell containing protein platelets (white arrows).

Bright yellow, late-stage embryos of ca. 180 μm in diameter (Fig 1B) were found scattered within the entire mesohyl in similar stages of development, right before the larval formation, in the four different analysed specimens (Table 1). The follicle of the embryo was a monolayer of flattened pinacocyte-like cells of an approx. 5 μm in width (Figs 3B, 6A–6C). Follicle cells extended pseudopodia both to the embryo and to the mesohyl in order to capture and transfer nutritional elements to the embryo (Fig 6A and 6B). In some regions, the follicle cells were bifurcated and contained extensive smooth endoplasmic reticulum and granular content similar to glycogen (Fig 6A and 6B). Beyond the follicle and outside the embryo, a thick layer of collagen (2–3 μm) was observed to structure and give consistency to the follicle (Fig 6A, 6B and 6D). The nurse cells were amoeboid (5–10 μm in max. diameter), forming pseudopodia to capture nutritional elements (Fig 6A, 6B, 6F and 6G). They were ingesting and digesting bacteria, among other material (Fig 6F), which they processed in vesicles and transformed into heterogeneous yolk protein platelets and glycogen (Fig 6F and 6G).

An unequal cleavage was observed in the embryos of *P*. *areolatus*, producing blastomeres of different sizes, being posterior micromeres 10 μm in max. length and anterior macromeres up to 35 μm (Figs 3B, 6D and 6E). The blastomeres had a nucleolated nucleus of ca. 5 μm in maximum length, occupying half of the cell body (Fig 6E). Blastomeres in the periphery of the embryo and close to the follicle layer were slightly smaller and more flattened than those of the internal part of the embryo which had a more spherical shape (Fig 6B and 6D). The blastomeres contained well-developed Golgi apparati heavily engaged in lipid formation (Fig 6E). Blastomeres contained large, rounded, lipid droplets, covering one third of the total blastomere area, and many smaller lipid droplets indicative of recent formation (Fig 6B, 6D and 6E). No bacteria were detected within the embryos.

### Oocyte and embryonic features in *Kirkpatrickia variolosa* (Demospongiae, Poecilosclerida, Hymedesmiidae)

**Fig 7:**
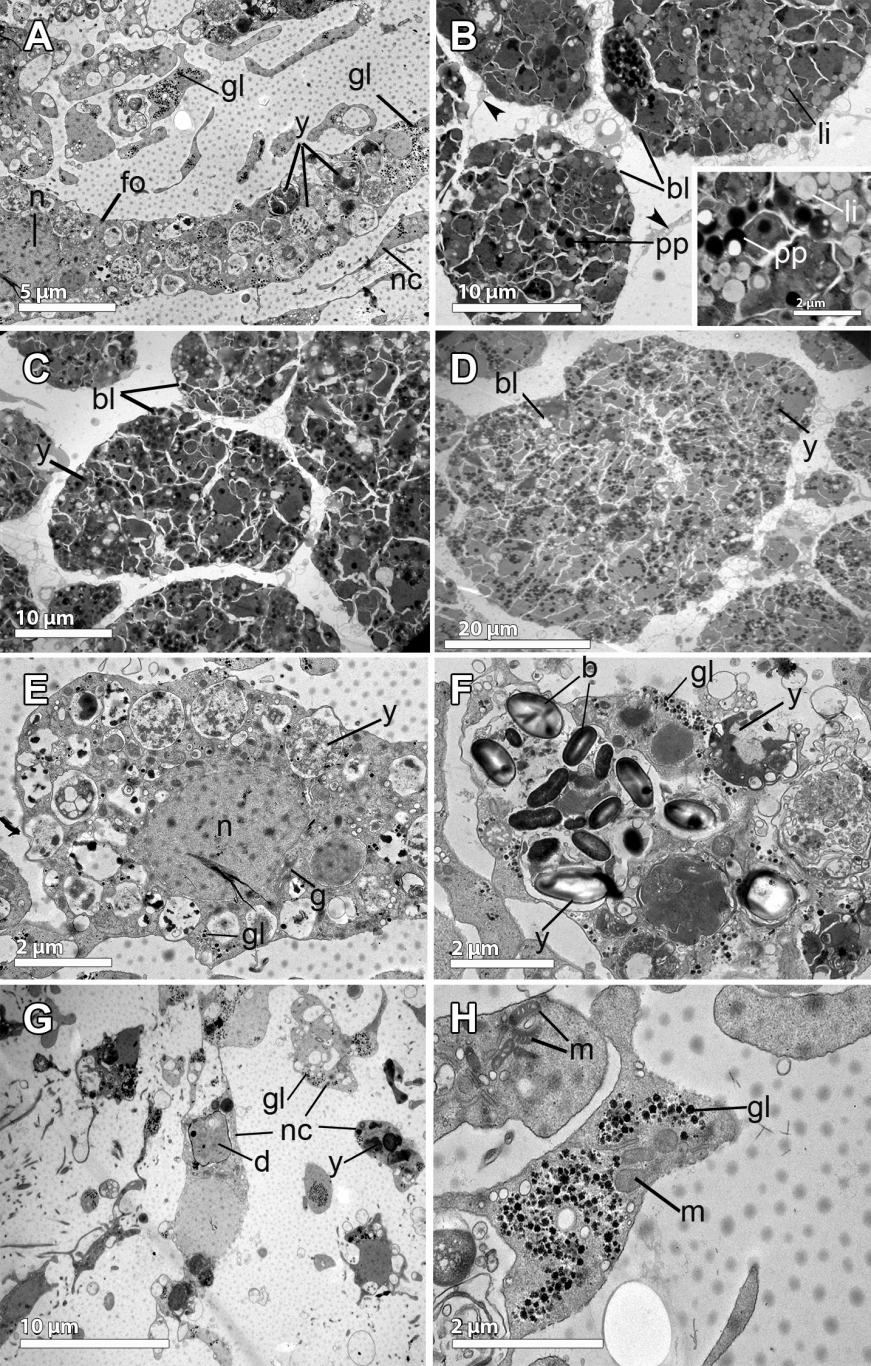
Ultrastructural features of the embryo of *Kirkpatrickia variolosa*. **A.** Monolayer of follicle cells (fo) containing multiple yolk platelets in different stages of yolk formation (y) and granules of glycogen (gl). Amoeboid-shaped nurse cells (nc) located in the periphery of the embryo. Note the nucleus (n) of the follicle cell. **B**. Micromeres (bl) of the posterior part, ca. 10 μm in diameter, and full of lipids (li) and protein yolk (pp). Projections of the blastomere cytoplasm indicated by black arrowheads. **Inset.** Close up of the cytoplasm of a blastomere showing lipid droplets (li) and protein platelets (pp). **C-D.** Anterior macromeres (bl), 20–50 μm in diameter, showing heterogeneous yolk platelets (y). **E.** Follicle cell showing the nucleus (n) and Golgi apparatus (g), heterogeneous yolk platelets (y) and glycogen (gl). **F-H.** Nurse cells dispersed in the mesohyl, some showing engulfed (or produced) material of different sources: unknown material, which could be interpreted as phagocytosed bacteria (b, within the vesicle of Fig 6F), diatoms (d), yolk (y) and glycogen (gl). Mitochondria (m) visible within the nurse cells.

Very few and small (ca. 21 μm in diameter) pre-vitellogenic oocytes were observed close to the canals of two out of the three studied individuals of *K*. *variolosa* (Fig 3C; Table 1). Bright yellow embryos (ca. 250 μm in diameter) were also observed within the body in several developmental stages (Figs 1C and 2), in much larger numbers than in *D*. *antarctica* and *P*. *areolatus* (Figs 1C insert, 3D). The follicle of the embryo was a monolayer of vesicular cells, ca. 5 μm in max. width (Fig 7A). Within the follicle cells, there were large amounts of glycogen and multiple vesicles containing several stages of yolk formation (Fig 7A). A large number of amoeboid nurse cells were located in the periphery of the embryo, in close proximity to the follicle (Fig 7A–7H). The nurse cells were non-nucleolated cells of 5–10 μm max. length (Fig 7A–7H), which engulfed including bacteria and diatoms (Fig 7F and 7G), which then were digested and transformed into yolk platelets (Fig 7F) to be later transferred to the embryo.

Early stage embryos (4–8 cells) were observed developing within the mesohyl (Fig 3D, insert) while mid- (200 μm) and late-stage (300 μm) embryos were present in the vicinity of the canals (Fig 3E and 3F). Embryonic blastomeres were the result of an unequal cleavage (Fig 3E and 3F). In mid- and late-stage embryos, posterior micromeres were 5–10 μm and anterior macromeres 20–50 μm (Figs 3E, 3F, 7B and 7D). The blastomeres were full of heterogeneous yolk platelets of both lipid and protein nature (Fig 7B–7D). Given their extreme yolky nature, it was difficult to observe the nucleus. Interestingly, blastomeres seemed to be connected by thin projections of the cytoplasm (Fig 7B). No bacteria were detected within the embryos.

### Embryonic features of *Isodictya kerguelenensis* (Demospongiae, Poecilosclerida, Isodictyidae)

**Fig 8:**
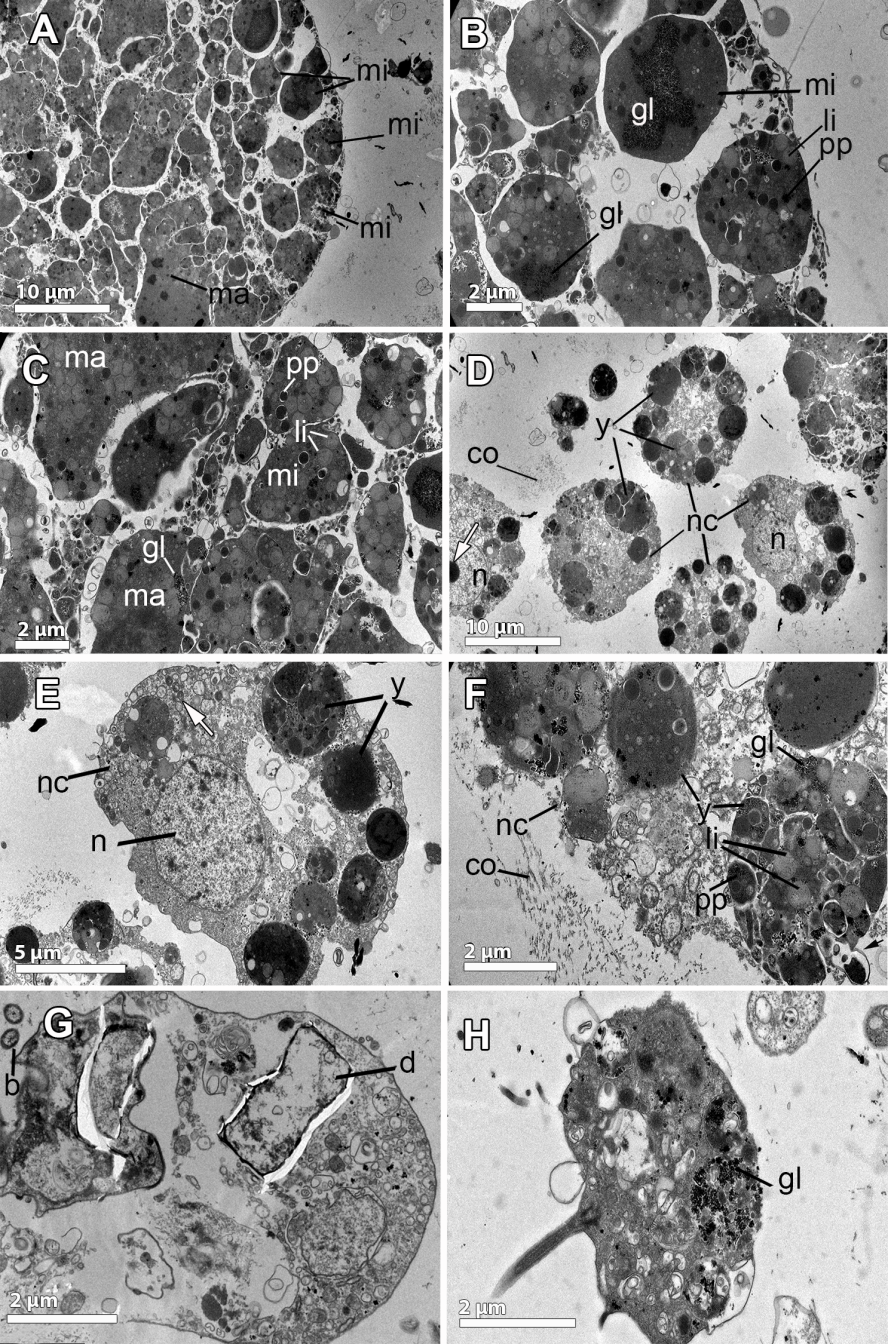
Ultrastructure of the embryo and nurse cells of *Isodictya kerguelenensis*. **A-B.** Embryo showing large macromeres (ma) and micromeres (mi), both with heterogeneous lipid (li) and protein (pp) yolk, and glycogen (gl). **C.** Micromeres (mi) and macromeres (ma), full of lipids (li), protein (pp) yolk, and glycogen (gl). **D.** Round nurse cells (nc) with nucleolated (white arrow) nuclei (n) within the mesohyl intermingled with collagen fibrils (co). Heterogeneous yolk platelets (y) within the cytoplasm of the nurse cells. **E-F.** Details of nurse cells (nc): large nucleus (n), heterogeneous yolk platelets comprised by lipid (li), protein (pp) and glycogen granules (gl). Mitochondria are abundant in the cytoplasm (white arrow). A collagen (co) layer in the mesohyl out to the nurse cells. Phagocytotic activity indicated by a black arrow. **G.** Phagocytosed diatoms (d) within nurse cells. Note the bacteria (b) within the mesohyl. **H.** Choanocyte showing large amounts of glycogen and heterogeneous yolk (y).

Very large (ca. 1 mm in largest diameter) red embryos were observed within the body in one out of the three analysed specimens of *I*. *kerguelenensis* (Figs 1D and 2; Table 1). The embryogenesis of this species showed several peculiarities. In particular, the embryonic follicle was not observed in our preparations (see Fig 8A), which could be due to the advanced developmental stage of the embryos or maybe due to sample preservation artifacts. The nurse cells were situated in the mesohyl very close to the developing embryos (Fig 8D), with a 10–15 μm max. diameter, and possessing a distinctive non-nucleolated nucleus that occupied a third of the cell area (Fig 8D and 8E). In each nurse cell, numerous heterogeneous yolk platelets were observed, measuring 0.5–7μm (Fig 8D–8F). Lipid, protein and glycogen granules were observed within the yolk granules (Fig 8D–8F). The nurse cells had an intense phagocytotic activity, and were observed ingesting bacteria (not shown) and diatoms (Fig 8G). Embryos were located in close vicinity of the choanocyte chambers, which possessed choanocytes with large accumulations of glycogen (Fig 8H). Symbiotic bacteria could be observed in the mesohyl (Fig 8G), although not in large numbers; no bacteria were observed within the embryos.

As in the previous poecilosclerids studied here, cleavage was unequal, producing large macromeres of 30–60 μm length (Fig 8A and 8B) and small posterior micromeres of 5–10μm (Fig 8A and 8B). Each blastomere contained numerous heterogeneous yolk granules of different sizes (1–8 μm) with lipid and protein material (Fig 8A–8C), being the lipids the dominant component. Within the blastomeres, large accumulations of glycogen were observed (Fig 8B).

### Oocyte and embryo features of *Hemigellius pilosus* (Demospongiae, Haplosclerida, Niphatidae)

**Fig 9:**
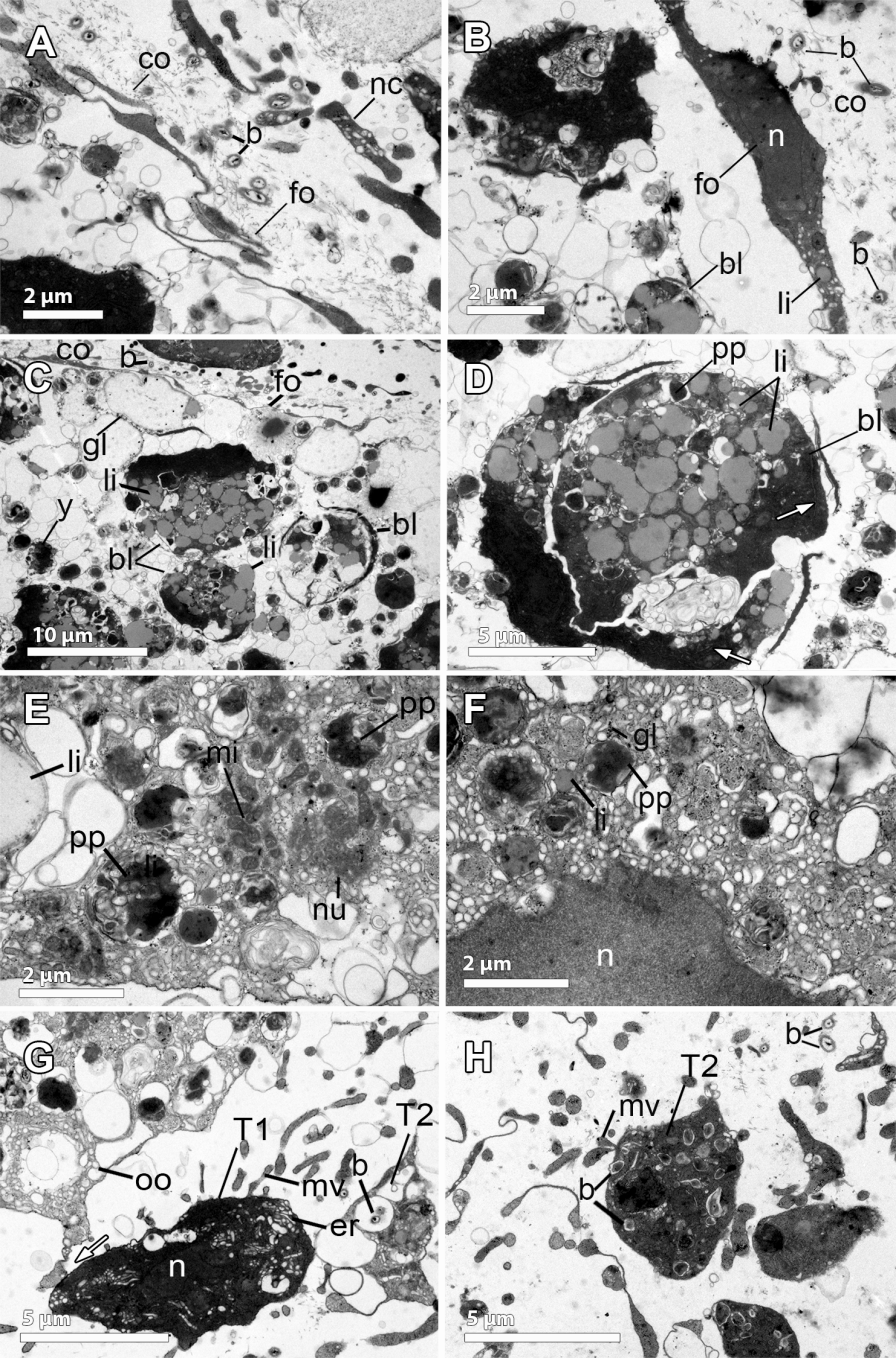
Ultrastructure of the reproduction in *Hemigellius pilosus*. **A-B.** Flattened follicle cells (fo), similar to pinacocytes, showing a distinct nucleus (n) and lipid droplets (li), surrounding the embryo (bl). In the mesohyl and close to the follicle, a loose collagen layer (co), bacteria (b) and nurse cells (nc) are observed. **C.** Blastomeres (bl), ca. 10 μm in diameter, with large numbers of lipid droplets (li), electron-dense yolk (y), and glycogen granules (gl). Note the follicle cells (fo) and the collagen layer (co) with bacteria (b) lying on it. **D.** Detail of blastomere showing the electron-dense cytoplasm (white arrow) containing great amount of lipid droplets (li) and protein yolk granules (pp). **E-F.** Details of previtellogenic oocytes showing the nucleus (n), heterogeneous yolk (y) platelets in different formation stages, protein (pp) platelets and lipid (li) droplets. Mitochondrial clouds (mi), arranged surrounding a granular structure similar to the nuage (nu). **G-H.** Nurse cells: Type 1 (T1) nurse cell, with large endoplasmic reticulum (er) and type 2 (T2) cells, engulfing and digesting bacteria (b) to produce yolk. Microvilli (mv) from nurse cells projected towards the mesohyl. Note the cell projection (white arrow) of the oocyte (oo) in direct contact with the nurse cell (T1).

Both oocytes and embryos were found within the three collected individuals of *H*. *pilosus* (Fig 3G–3I; Table 1). Pre-vitellogenic oocytes, not enclosed by a follicle, were found close to the canals (Fig 3G). Oocytes were ca. 80 μm in max. diameter (Fig 2). They possessed an eccentric nucleolated nucleus (Fig 3G); the ooplasm contained several heterogeneous yolk platelets in different stages of formation (Fig 9E and 9F), with large lipid droplets surrounded by a membrane in the periphery and with several mitochondrial clouds (Fig 9E). The mitochondria were arranged surrounding a granular structure similar to the nuage (Fig 9E). The oocytes contained phagocytosed nurse cells within their ooplasm (Fig 3G). Two types of nurse cells were observed near the oocytes: type 1, with large endoplasmic reticula (Fig 9G) and type 2, engulfing and digesting bacteria to produce yolk (Fig 9G and 9H). Both projected numerous microvilli towards the mesohyl (Fig 9G and 9H). Direct contact of the nurse cell microvilli and the oocyte membrane was observed (Fig 9G).

Embryos in different stages of the development were found scattered throughout the entire mesohyl (Figs 1E, 3H, 3I, 9A and 9D) with 220 μm average max. diameter (Fig 2). All embryos were enveloped by a follicle (Figs 3H, 3I, 9A and 9C) that was a thin monolayer of cells of ca. 0.5μm of largest width (Fig 9A–9C). Follicle cells were flattened pinacocyte-like cells with a distinct nucleus and contained lipid droplets (Fig 9B). Follicle cells were intertwined in most cases although, sometimes, a “loose end” could be observed (Fig 9C). In the outer part of the follicle, a relatively loose 2 μm-layer of collagen was observed with several bacteria (Fig 9A–9C). Follicle cells extended microvilli to phagocyte particles within the mesohyl (Fig 9B) to be later transferred to the embryo.

Early embryos were ca. 200 μm in average diameter (Fig 3H), while late embryos were larger, 300 μm of largest diameter (Fig 3I). Embryos showed equal cleavage (Fig 3H and 3I) and blastomeres varied from 15μm of largest diameter in early embryos (Fig 3H) to 50 μm of largest diameter in late embryos (Fig 3I). Blastomere cytoplasm was very electron-dense, having a great amount of lipid droplets and also protein yolk granules (Fig 9C and 9D) as well as a well-developed endoplasmic reticulum, which was difficult to observe given the dark cytoplasm (Fig 9D). Interestingly, some blastomeres seemed to be engulfing other blastomeres, or at least they were observed projecting cells extensions to surround other blastomeres (Fig 9C and 9D). Large empty vesicles were observed within the embryo, probably result of the digestion of lipid content (Fig 9D).

### Oocyte and embryonic features of *Haliclona penicillata* (Demospongiae, Haplosclerida, Chalinidae)

**Fig 10:**
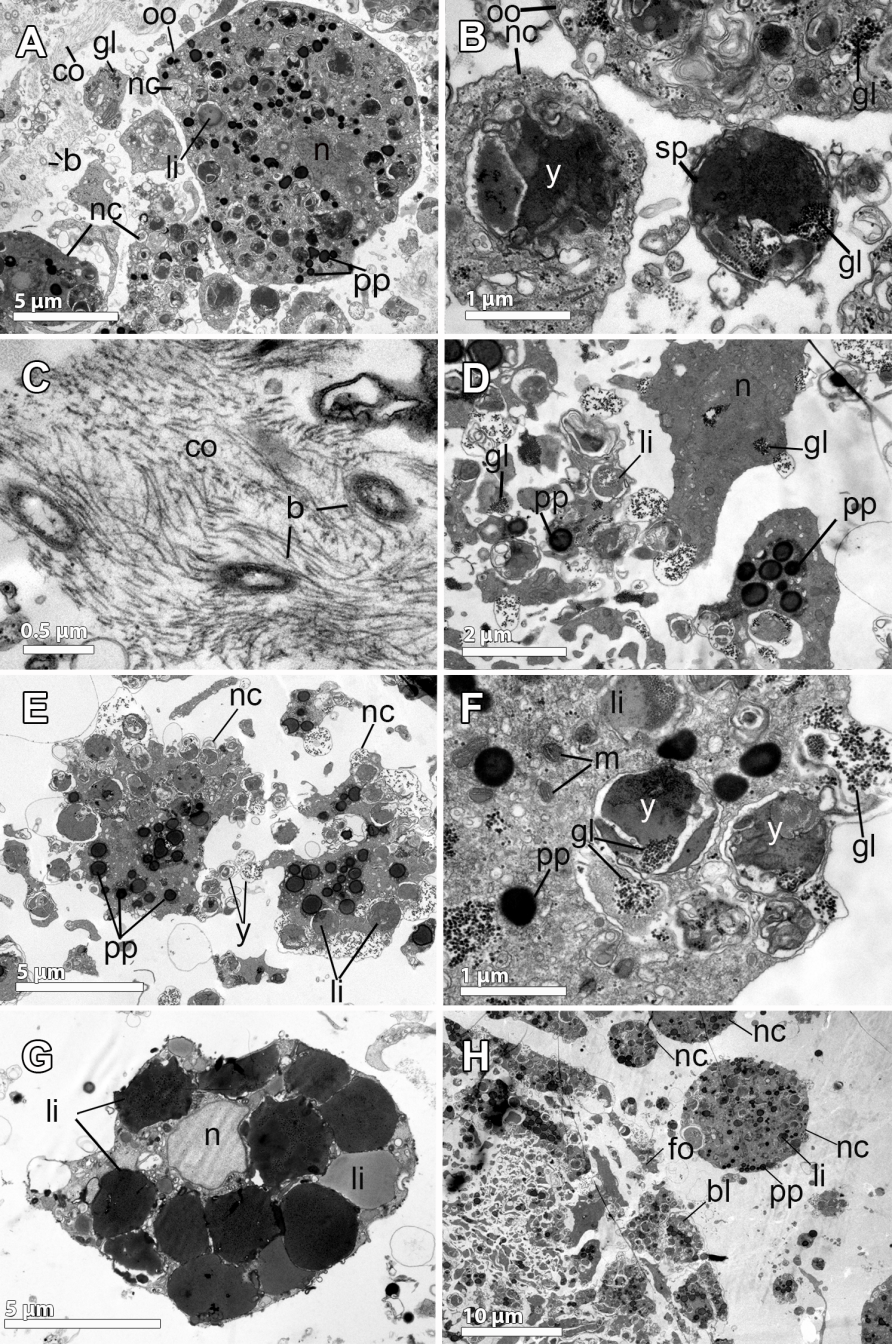
Ultrastructure of the reproductive elements in *Haliclona penicillata*. **A.** Oocyte (oc) in the mesohyl with a distinct nucleus (n), yolk platelets (pp) and lipid droplets (li). Nurse cells (nc) with glycogen (gl) and a collagen layer (co) observed within the mesohyl and in close proximity of the oocyte. Bacteria (b) lying on the collagen layer. Engulfed nurse cells (nc) within the oocyte. **B.** Detail of a nurse cell close to the oocyte containing yolk (y). A possible spermatozoon (sp), ca. 1 μm in diameter, with a condensed nucleus and glycogen (gl) in close proximity to the oocyte. **C.** Detail of the collagen layer in the mesohyl where the oocytes were present. Note the bacteria lying among the collagen. **D.** Amoeboid small blastomeres with nucleus (n), ca. 2 μm in diameter, containing glycogen (gl), protein (pp) and lipid (li) yolk. **E-F, H.** Nurse cells (nc) close to the embryonic follicle (fo) with heterogeneous yolk granules (y), protein platelets (pp) and lipid droplets (li). Glycogen (gl) and numerous mitochondria (m) within the cytoplasm. **G.** Large amoeboid cell located close to the embryo, ca. 10 μm in diameter, characterized by a large non-nucleolated nucleus (n) and large amounts of different electron-density lipid droplets (li).

Both oocytes and cleaving embryos were observed within the tissue of two of the three collected individuals of *H*. *penicillata* (Table 1), indicating that fertilization does not take place synchronously in this species (Figs 3J, 3K, 10A and 10E). Many vitellogenic oocytes were located within the mesohyl and had an average size of 20 μm (Figs 2, 3J and 10A). They contained lipid and protein yolk platelets and also glycogen (Fig 10A and 10B) as well as engulfed nurse cells in which the nuclei was no longer visible (Fig 10A). Small nurse cells (2–5 μm) that contained yolk of similar appearance of that of the oocyte, were observed surrounding the oocyte (Fig 10B) and with a general appearance similar to that of engulfed nurse cells within the oocyte (Fig 10A). The mesohyl, where oocytes were observed, presented a 1 μm-layer of collagen filled with bacteria (Fig 10A–10C). In close proximity to the oocyte, a possible spermatozoan was observed (Fig 10B). It possessed a small round body (ca. 1 μm) with a highly condensed nucleus that occupied half the cell body (Fig 10B). Glycogen was also detected within the cell body of the putative spermatozoan (Fig 10B). The oocytes were not surrounded by a follicle (Figs 3J and 10A), but the embryo was enveloped by a very thin cellular follicle of flattened cells (Figs 3K and 10H) that sometimes was not visible.

Embryos were observed in brooding chambers, which contained between 30–60 embryos each (Fig 1F). Embryos were relatively small, between 75–100 μm in largest diameter (Fig 2K). Inside, two cell types were observed: small round to amoeboid cells of 2 μm in largest diameter (Figs 2K, 10C and 10D) and large cells of 20–30 μm in largest diameter (Fig 2K). All embryonic cells contained heterogeneous yolk platelets, including protein yolk and large amounts of glycogen (Figs 2K, 10C, 10D and 10F). Interestingly, large cells contained heterogeneous yolk platelets in different stages of formation, showing that glycogen was packed with material of lipid appearance into platelets of ca. 0.5 μm (Fig 10F). The round large embryonic cells were similar in shape and content to the nurse cells (Fig 10E and 10H), which had also protein yolk and heterogeneous platelets, and could have been engulfed via phagocytosis. In addition, larger cells containing large amounts of lipid droplets of different electron-density were observed close to the embryos (Fig 10G). High phagocytosis activity and transfer of nourishing material were observed among all types of cells inside the embryo (Fig 10C and 10D).

## Discussion

### General considerations of sexual reproduction in the species under study

All species reported in this study were brooding their embryos within their mesohyl (Table 2), like their hymedesmiid [22–26], niphatid [25,27,28], chalinid [19, 27,29–34], darwinellid [35], and isodyctid [6] counterparts of the same and other latitudes. Even though all members of the family Hymedesmiidae have been reported as hermaphrodites [22–26] male reproductive features were not observed in any of the studied specimens of *P*. *areolatus* nor *K*. *variolosa*. Although this could be related to the relatively small sampling size in our study, it could also indicate a successive production of different gametes within an individual, with spermatogenesis preceding oogenesis as in other sponges [19]. Our sampling took place during the austral summer (where sea temperature is highest). In cold-water species of Chondrillida and Poecilosclerida [36], spermatogenesis has been reported during early spring, followed by maturation of oocytes and embryogenesis in early and late summer. Like in *P*. *areolatus* and *K*. *variolosa*, no sperm was detected in *D*. *antarctica*, *I*. *kerguelenensis*, *H*. *pilosus* and *H*. *penicillata*. However, the absence of male reproductive features in these cases could be due to a biased sampling of only females (which are usually much more abundant), since most of the members of the respective families are gonochoristic, except for some exceptions such as *Haliclona (Haliclona) oculata*, *Haliclona (Soestella) xena* [32], *Haliclona (Haliclona) varia* [31] and *Niphates nitida* [27]. The reproductive strategy of Dendroceratida is only known for a few species [35,37–42], proposing a simultaneous hermaphroditism for this group. Nonetheless, only female or male gametes were found in the species where gametogenesis was studied, and never both at the same time [38–40, 42].

**Table 2:** Summary of reproductive features observed in all studied species.

Species	*Dendrilla antarctica*	*Phorbas areolatus*	*Kirkpatrickia variolosa*	*Isodictya kerguelensis *	*Hemigellius pilosus*	*Haliclona penicillata*
**Order**	Dendroceratida	Poecilosclerida	Poecilosclerida	Poecilosclerida	Haplosclerida	Haplosclerida
**Oocytes**	34 μm, isolecithal, vitellogenic	Not found	22 μm, isolecithal, pre-vitellogenic	Not found	80 μm, isolecithal, pre-vitellogenic	20 μm, isolecithal, vitellogenic
**Spermatozoa**	Not found	Not found	Not found	Not found	Not found	putative (1 μm)
**Embryos**	Yellow ca. 140 μm	Bright yellow Late stage: ca. 180 μm	Bright yellow Early-stage: ca.100 μm Mid-stage: ca. 200 μm Late-stage: ca. 300μm	Red Late stage: 1 mm	White Earl-stage: 200 μm Late-stage: 300 μm	White ca. 75–100 μm
**Blastomeres**	20–30 μm	Micromeres: 10 μm Macromeres: 35 μm	Micromeres: 5–10 μm Macromeres: 20–50 μm	Micromeres: 5–10 μm Macromeres: 30–60 μm	From 15 μm (early) to 50 μm (late)	From 2 μm to 20–30 μm
**Cleavage**	Equal	Unequal	Unequal	Unequal	Equal	Unequal
**Follicle**	Monolayer of flattened pinacocyte-like cells	Monolayer of flattened pinacocyte-like cells	Monolayer of vesicular cells	Not observed	Thin monolayer of pinacocyte-like cells	Thin monolayer of flattened cells
**Nurse cells**	Amoeboid (ca. 5 μm max. diameter), in the mesohyl	Amoeboid (ca. 5–10 μm max. diameter), in the mesohyl	Amoeboid (ca. 5–10 μm max. diameter), in the mesohyl	Type 1 and type 2 (ca. 10–15 μm max. diameter), in the mesohyl	Type 1 and type 2 (ca. 5 μm max. diameter), within oocytes and in the mesohyl	Amoeboid (ca. 2–10 μm), within oocytes and embryos, in the mesohyl
**Type of Yolk**	Heterogeneous	Heterogeneous	Heterogeneous	Heterogeneous	Heterogeneous	Heterogeneous
**Origin of Yolk**	Homosynthesis/Heterosynthesis	Heterosynthesis	Heterosynthesis	Heterosynthesis	Heterosynthesis/ Heterosynthesis	Heterosynthesis/ Heterosynthesis

Interestingly, a cell resembling a spermatozoan was observed close to an unfertilized egg in *H*. *penicillata* (Table 2). Fertilizing spermatozoans in sponges have only been reported for calcareous and homoscleromorph species, either in choanocyte chambers or being carried to the oocyte by a carrier cell [21,43], but never loose in the mesohyl as in *H*. *penicillata*.

### Sexual reproductive features in Dendroceratida

The reproductive elements of members of Dendroceratida have been reported only for a handful of species, being male gametes reported more often than oocytes [35,37–42]. Oocytes have been found for several species, including *Dendrilla rosea*, *Dictyodendrilla dendyi* [40] and *Aplysilla sulfurea* [42], but the complete oogenesis has only been completed in *A*. *sulfurea*, finding asynchronous developing oocytes scattered throughout the choanosome [42]. Our observations in *D*. *antarctica*, however, pointed to a synchronic oogenesis in the analysed individuals. Both the vitellogenic oocytes of *D*. *antarctica* and those in *A*. *sulfurea* [42] were isolecithal (Table 2), located close to the canals and were surrounded by a thin cellular follicle, with a relatively large space between the oocyte and the follicle. However, no phagocytosed nurse cells were found within the vitellogenic oocytes of *D*. *antarctica* (Fig 3A), as observed for *A*. *sulfurea*, suggesting that the yolk is only produced by the oocytes themselves and also transferred to them from the surrounding nurse cells.

As said, the embryogenesis of *D*. *antarctica* was synchronic within the individual in contrast to what happens in *A*. *sulfurea* [42], which indicates a single sperm spawning event within the populations. A single sperm spawning in this sponge species could be an adaptation to very short-term favourable conditions, typical of Antarctic regions. In *D*. *antarctica* as in *A*. *sulfurea*, the cleavage was total and equal (Table 2), but the late stage embryos were smaller in *D*. *antarctica* [42]. Interestingly, the embryos of *D*. *antarctica* had spongin fibres located among the blastomeres, something that has only been reported previously in the larvae of *A*. *sulfurea* [42]. Most of the yolk in *D*. *antarctica* was heterogeneous, similar to that reported in oocytes of *A*. *sulfurea* [42]. In regard to the embryo, it is important to highlight that yolk has never been investigated for any dendroceratid, to our knowledge, and thus our study is the first to report homosynthesis of lipid yolk in a dendroceratid species (Table 2).

### Sexual reproductive features in Poecilosclerida

Early pre-vitellogenic oocytes were found only in the choanosome of *K*. *variolosa*, close to exhalant canals (Fig 3C). Although oocytes have been reported within choanocyte chambers in other poecilosclerids [44], we just found them lying in the choanosome between choanocyte chambers. No follicle, either collagenous or cellular, was observed surrounding the oocyte in *K*. *variolosa*, although some cells have been observed chaotically arranged around the oocytes of other poecilosclerids [26,44–45].

In *P*. *areolatus* and *I*. *kerguelensis*, the embryos found in the choanosome were in similar developing stages (Fig 3B–3F) but in *K*. *variolosa* we found embryos in early-, mid-, and late-stages (Fig 3E and 3F). As in *D*. *antarctica*, this pattern of synchronic embryo development suggests single episodes of sperm spawning, potentially coupled with the early Antarctic Summer. The embryos of *P*. *areolatus* and *K*. *variolosa* were relatively similar in size (170–250 μm) to those previously reported for congeneric species from the Mediterranean [42]. The embryos of *I*. *kerguelenensis*, previously reported to be slightly smaller (ca. 800 μm in largest diameter; [6] were similar in size to those of another Antarctic isodictyid, *I*. *setifera* [6], being among the largest embryos ever reported in sponges. The closest largest embryos (ca. 700 μm in largest diameter) were reported for the Antarctic suberitid *Stylocordyla chupachups* [8]. To our knowledge, there is no information available on the embryo sizes for any isodictyd or suberitid from temperate habitats that could be used for comparison. This large size reported in embryos for Antarctic sponge species is not unusual, since other Antarctic invertebrates present ‘gigantic’ eggs when compared to their counterparts from temperate habitats [46,47]. One of the reasons that could explain such gigantism could be the high oxygen availability in polar oceans [48]. Also, phylogenetic constraints might have a role in determining the size of the embryos. The family Isodictydae is basal within Poecilosclerida, more closely related to the Crambeidae [49], which usually have larger larvae [6,50] than the rest of the poecilosclerids, e.g. [24–26].

Embryogenesis in the three investigated poecilosclerids progressed through unequal cleavage (Table 2), as in all poecilosclerids studied so far [15,26], except for the carnivorous sponge *Lycopodina occidentalis* where cleavage was equal [21]. The macromeres in our target species were observed in the central part of the embryo while the micromeres were surrounding the macromeres, as it is usual for other poecilosclerids [15,26].

The follicle in poecilosclerids is sometimes “unusual”. In most instances, it is comprised by one or more layers of flattened cells that aid in embryo nurture, surrounded by a collagen layer [e.g. 26], that in some cases is secreted by the embryo [51–55]. However, for the Antarctic *Tedania (Tedaniopsos) charcoti* and *Tedania (Tedaniopsis) tenuicapitata* the follicle seems to disappear after the embryogenesis is completed and is replaced by spicules [6]. Even though we observed the disappearance of the follicle in *K*. *variolosa*, no spicules were found replacing it. Our observations of the bifurcated cells in the follicle of *P*. *areolatus* are intriguing since despite most poecilosclerids have a well-developed follicle in the oocyte and embryo [15], this is always built with single, non-bifurcated cells.

### Sexual reproductive features in Haplosclerida

Oocytes and embryos in the chalinid *H*. *penicillata* and the niphatid *H*. *pilosus* were observed during the Antarctic summer. There are very few previous reports of sexual reproduction in Antarctic haplosclerids [6,7]. Early stage oocytes and embryos were reported for *Haliclona bilamellata* in the SubAntarctic South Georgia Island during the Antarctic winter [6] while large embryos were observed during the Antarctic summer in *Pachypellina fistulata* from the Antarctic Peninsula [7].

Haplosclerids usually brood their embryos and larvae, which are sometimes scattered throughout the entire mesohyl as in *H*. *pilosus*, e.g. [56–59] and sometimes in brood chambers as in *Haliclona penicillata*, e.g. [58,60]. Niphatid and chalinid sponges are reported to be either hermaphroditic [e.g. 25,32,60] or gonochoric [e.g. 19,30,59]. Even though we did not find any male reproductive element (Table 2), we cannot rule out that *H*. *pilosus* and *H*. *penicillata* were sequential hermaphrodites. Further studies on these two species by collecting samples in other periods of the year are strongly needed to clarify this.

The oocytes of haplosclerids very often contain engulfed nurse cells, like those found in *H*. *pilosus* and *H*. *penicillata* [19,30,42,59,60] which provide yolk to the developing oocyte. In our case, the nuclei of the nurse cells are indistinguishable once they are engulfed by the oocyte, similar to the process reported in *Chalinula ecbasis* and *Chalinula loosanoffi* [19,30,61] and *Haliclona* (*Gellius) angulatus* [62]. In other chalinids though, the nucleus remains intact [15], while in most studied haplosclerids the entire cytoplasm is filled by nurse cells (see [15] for a review).

Cleavage in haplosclerids is considered to be unequal [61], however, most data come from freshwater spongillids, previously considered to be haplosclerids but now belonging to a separate order, Spongillida [63]. *H*. *penicillata* showed unequal cleavage as the Mediterranean *Haliclona (Haliclona) simulans* [22], although other species such as *Chalinula ecbasis* are reported to have equal cleavage [61]. In any case, given the amount of nurse cells and yolk in the haplosclerid embryos, the cleavage is difficult to follow and further detailed studies should be conducted to establish the patterns of embryonic cleavage in this group.

### Ecological implications of the reproductive patterns of Antarctic demosponges

Demosponges are the most common and conspicuous organisms in Antarctic waters [64], which is especially true for the shallow-waters of the South Shetland Islands and Antarctic Peninsula [4]. Although all studied species were collected in the same area and thus share the same habitat, they have developed slightly different reproductive traits likely to succeed in their coexistence avoiding direct competition between them. While the reproductive periods of all species coincide in time, it appears that some of them rely on a single event of sperm release for the fertilization phase, indicated by the occurrence of embryos in very similar stages of development (as in *P*. *areolatus* and *I*. *kerguelenensis*). In turn, other species seemed to stagger their sperm release in time (as in *H*. *penicillata*), which could suggest a strategy for taking advantage of more opportunities for larval release and therefore successful settlement, as it was suggested for Mediterranean sponge species [54].

Reproductive features such as timing, content and quantity of nutrient reserves are indicators of the developmental stage of the embryo/larvae, and provide information about their reproductive strategy, and ultimately its potential dispersal [9,26,51]. While it is common that sponge embryos and larvae contain a mix of different energetic sources, including lipid/protein yolk and glycogen [9,21,26,65], the embryos of the Antarctic *Mycale (Oxymycale) acerata* have been reported to have much higher content of lipid yolk than its tropical counterpart *Mycale (Mycale) laevis* [9]. Protein synthesis requires a higher energetic demand than lipid production [66], and in cold waters especially, protein synthesis is even more costly than in other habitats [67,68]. However, we did not observe such differences in the amount and nature of the yolk in the species studied herein, and, therefore, we believe that the higher investment in lipid yolk during the embryogenesis of *M*. *(Oxymycale) acerata* might be a specific adaptation rather than a trend related to the environmental pressures of the Antarctic ecosystem. Further studies will be conducted in congeneric species inhabiting contrasting thermal regimes to assess whether a specific production of any yolk type might be related to the energetic requirements of sponges of higher or lower temperatures.
